# Cervical Epidural Anaesthesia for Radical Mastectomy and Chronic Regional Pain Syndrome of upper limb-A Case Report

**Published:** 2009-12

**Authors:** Ashok Jadon, Prashant S Agarwal

**Affiliations:** 1 Sr. Consultant & HOD Anaesthesia; 2 DNB Trainee Anaesthesia

**Keywords:** Cervical epidural anaesthesia (CEA), CRPS (chronic regional pain syndrome), Carcinoma breast, epidural Clonidine

## Abstract

**Summary:**

A 47-yrs-female patient presented with carcinoma right breast, swelling and allodynia of right upper limb. radical mastectomy with axillary clearance and skin grafting was done under cervical epidural anaesthesia through 18G epidural catheter placed at C6/C7 level. Postoperative analgesia and rehabilitation of affected right upper limb was managed by continuous epidural infusion of 0.125% bupivacaine and 2.5 µg/ml^−1^clonidine solution through epidural catheter for 5 days and physiotherapy. This case report highlights the usefulness of cervical epidural analgesia in managing a complex situation of carcinoma breast with associated periarthitis of shoulder joint and chronic regional pain syndrome (CRPS) of right upper limb.

## Introduction

Mastectomy for carcinoma breast is usually done under general anaesthesia. However, there is growing interest to do this surgery under regional (cervical epidural or upper thoracic epidural) anaesthesia. The advantages are less intra-operative blood loss, less intra-operative surgical stress and better postoperative analgesia^1^,^2^. All these factors help to decrease morbidity and mortality in such patients. The chronic regional pain syndrome; CRPS (type I & II) is a group of sympathetically mediated pain in different body parts and extremities (upper limb and lower limb) are commonly involved. The pain management of such patients is very difficult and often necessitates interventional pain management procedure. We have managed a case of carcinoma breast scheduled for mastectomy and also suffering from CRPS (type I). Mastectomy was done under cervical epidural anaesthesia and same epidural catheter was used to manage postoperative pain of surgery and pain due to CRPS of right upper limb. The cervical epidural was very effective to control her pain and helped in physical therapy also.

## Case report

**Fig 1:**
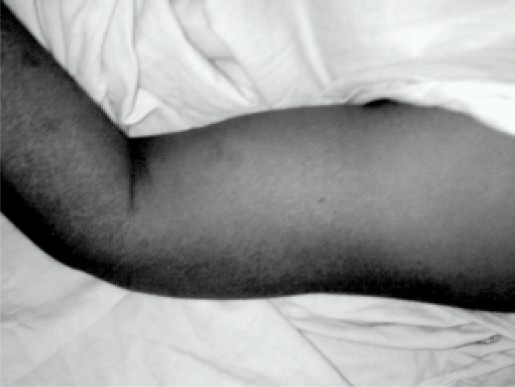
Right upper limb showing features of CRPS; swelling, changes in skin and hair loss.

A-47-yrs, female patient with carcinoma right breast was scheduled for right radical mastectomy and axillary clearance. She had history of severe pain and restriction of movements in right upper limb since 3 months. On examination affected limb showed signs of CRPS (chronic regional pain syndrome) e.g. swelling, skin changes, loss of hair (Fig-1) and allodynia (severe pain on slight touch or even with contact of cloths). There was history of trauma to right shoulder due to fall. There was no other significant medical history. General physical examination and routine investigations were with in normal limits.

Surprisingly her main complaints were pain in right shoulder (known patient of periarthitis right shoulder) and right upper limb for which she was consulting orthopedic surgeon. She also had complaint of insomnia and psychiatric disorder (depression) of recent origin. However, as she was not getting any relief in pain and other symptoms with analgesic and anxiolytic drugs and also, pain extended to right side chest she was referred to the surgeon. Hard lump in the right breast along with enlarged lymph nodes in axilla were noticed. FNAC was done which showed malignancy and mastectomy was planned.

## Anaesthesia Technique:

**Fig 2:**
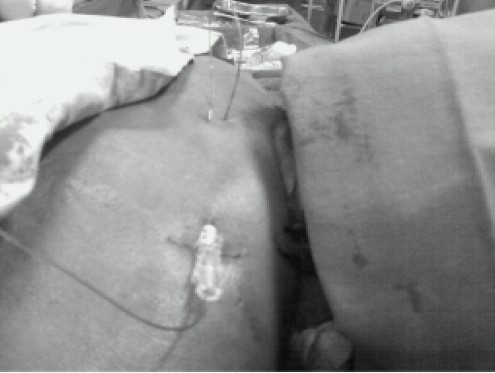
Cervical epidural catheter is being tunneled before transparent dressing.

**Fig 3:**
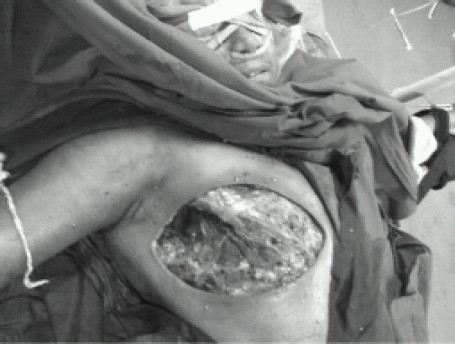
Shows dry surgical field after removal of breast and axillary clearance.

The epidural puncture at the C6/C7 intervertebral space was performed using the loss of resistance to saline technique via a paramedian approach in prone position using fluoroscopy. After location of the cervical epidural space, 18 G epidural catheter (Perifix^®^ 400 Filter set, B-Braun Medical (I) Pvt. Ltd) was advanced 4 cm cranially into the epidural space. Catheter was fixed by subcutaneous tunnel before adhesive dressing (Fig-2). The patient was made supine, and after checking vital signs, a test dose of 3ml 2% lidocaine was administered through catheter. Vital signs (breathing, consciousness, noninvasive arterial blood pressure, and electrocardiogram) were monitored for 5 min after the test dose and every 5 minutes there after till completion of surgery. After 5 minutes of test dose 7 ml 2% lidocaine was injected. The surgery was started after adequacy of sensory block/analgesia (C4-T10) was assessed with pinprick test. After 60 minutes 5ml 2% lidocaine was injected when patient was feeling pain during axillary clearance. During harvesting of skin grafts from right thigh, 50mg preservative free ketamine (Aneket^®^ Neon Laboratories Ltd) +5ml 2% lidocaine was injected through epidural catheter and grafts were taken without any discomfort. During surgery surgeon appreciated that operative blood loss was apparently less and surgical field was relatively dry (Fig-3). Surgery lasted for 180 minutes. After surgery patient was pain free, awake, having good muscle power in all four limbs and was without any clinically evident respiratory compromise (adequate res- piratory rate, depth and saturation).

Postoperative analgesia was provided with continuous epidural infusion of 0.08% bupivacaine and 2.5µg/ml^−1^clonidine (Cloneon^®^, Neon Laboratories Ltd.) solutions @ 2ml.hr^−1^. Vitals (pulse, blood pressure respiratory rate and SpO2) were monitored 2 hourly on day 1 and 4 hourly or SOS (when ever needed) from next day. Pain on rest and on movement was assessed by VAS (visual analogue scale) every hr on first day and 4 hourly next day onwards, when patient was awake. If VAS was 3 or more, and or patient demanded, 2–4 ml bolus of epidural injection of same solution being infused was given as rescue analgesic. Infusion was continued for 5days. Physiotherapy was started after 24 hrs. Patient had good postoperative pain relief and pain in arm was also controlled (>90% reduction) on hospital discharge. Patient did not have any complication either during surgery or in postoperative period. During follow-up patient was very satisfied, attitude was positive and had only slight discomfort in affected limb and was under regular physiotherapy.

## Discussion

Radical mastectomy for breast cancer is usually done under general anaesthesia (GA). However, there has been increasing interest in the use of cervical or high thoracic epidural anaesthesia due to economic reasons, less postoperative morbidity and excellent postoperative analgesia.^1^,^2^ Contrary to previous belief regarding propensity of complications with CEA (cervical epidural anaesthesia), it is now suggested equally safe alternative in surgery of breast, neck and upper extremity. 2,3 In our case CEA provided excellent surgical conditions, skin grafts were taken without supplementation of general anaesthetics (epidural ketamine with local anaesthetic was used), and there was appreciably less blood loss during surgery. Postoperative pain relief was also excellent (VAS remains 3 or less with 1 to 2 additional boluses with in 24 hrs, and patient had satisfactory sleep during hospital stay). The pain in right upper limb was also significantly decreased (VAS 4.33±1.6, during first 24 hrs and 3.3± 1.1 during rest of thedays till discharge form hospital).

Second most important consideration in our case is, whether she was really having CRPS or not, and the evidence to justify use of epidural clonidine to manage this condition. Complex regional pain syndromes (CRPS) are the most complicated chronic neuropathic pain syndromes with poorly defined etiology diagnosis and treatments. Past research indicates several causes linking to the disease and several risks factors for development of CRPS. Female gender, history of trauma, immobilization of part and psychological predisposition are important among the long list.^4^,^5^ Based on multicenter clinical study the following recommendations are currently proposed as diagnostic criteria.^6^

- Presence of at least two symptoms: Hyperesthesia, temperature and/or skin color changes, edema and/or sweating abnormalities, decreased range of motion, weakness and tremor.
- Presence of at least two signs: Allodynia and/ or hyperalgesia, objective temperature and skin color abnormalities, objective edema and/or sweating abnormalities, objective range of motion, weakness and tremor.

Our patient had all the components to qualify for clinical diagnosis of CRPS.

The pathophysiology of the disease is poorly understood and the treatment mostly based on anecdotal experience. Epidural clonidine injection is considered as an effective method of obtaining analgesia without serious complications.^7^,^8^ A double blind study has proven statistically significant pain relief of CRPS after epidural clonidine injection.^9^

Our patient also responded favorably to epidural clonidine.

Mobilization is integral part of CRPS management and epidural analgesia is one of the recommended techniques to attenuate pain of mobilization.^10^ In present case this was easily achieved due to infusion of local anaesthetic and clonidine mixture through epidural catheter. The clonidine is α_2_ agonist and it acts through modulation of autonomic nervous system. However, the exact pathophysiology of CRPS is unknown and the hypothesis that dysfunction of the peripheral autonomic nervous system plays important role in the development of CRPS is equally supported and criticized.^11^,^12^

We used clonidine infusion for 5 days without any side effect in our patient. We observed that epidural clonidine was safe and effective to manage a complex clinical syndrome like CRPS. Large case series also have proved that epidural clonidine is safe, effective and does not cause any serious side effects even if used for longer duration (4–7 weeks). 8 Cervical epidural anaesthesia can safely be used for radical mastectomy; it provides good operative conditions and postoperative analgesia. Clonidine and local anaesthetic mixture infusion through cervical epidural catheter can be used safely and effectively to mitigate the symptoms of CRPS of upper limb and to facilitate the mobilization.
